# Research evidence is essential for the development of family medicine as a discipline in the Japanese healthcare system

**DOI:** 10.3399/bjgpopen19X101650

**Published:** 2019-06-26

**Authors:** Makoto Kaneko, Ai Oishi, Yoshinori Matsui, Junichiro Miyachi, Takuya Aoki, Maria Mathews

**Affiliations:** 1 Assistant Professor, Department of Family and Community Medicine, Hamamatsu University School of Medicine, Hamamatsu, Shizuoka, Japan; 2 GP, Shizuoka Family Medicine Programme, Akatsuchi, Kikugawa, Shizuoka, Japan; 3 PhD Student, Primary Palliative Care Research Group, Usher Institute of Population Health Sciences and Informatics, The University of Edinburgh, Edinburgh, UK; 4 GP, Azai-higashi Clinic, Nagahama, Shiga, Japan; 5 GP, The Hokkaido Centre for Family Medicine, Hokkaido, Japan; 6 Assistant Professor, Department of Healthcare Epidemiology, School of Public Health in the Graduate School of Medicine, Kyoto University, Kyoto, Japan; 7 Professor, Department of Family Medicine, Schulich School of Medicine & Dentistry, Western Centre for Public Health and Family Medicine, London, ON, Canada

**Keywords:** Family medicine, health care system, Japan

Japan is facing an extraordinary rapid ageing rate: approximately 40% of people will be ≥65 years of age in 2060.^1^ The Japanese Ministry of Health, Labour and Welfare (MHLW) have highlighted the importance of primary care physicians for coping with the ageing population and reducing healthcare expenditure.^2^ However, a range of stakeholders such as MHLW and the Japan Medical Association (JMA) have been debating the necessity of family medicine as a medical discipline in the Japanese healthcare system. We examined the necessity of family medicine based on the existing discussion in the Japanese healthcare system and propose future research in this area.

The main characteristics of the healthcare system in Japan are universal health insurance and a free-access system, whereby patients are free to choose any healthcare facility, regardless of their insurance status or severity of illness.^3^ All residents of Japan including foreign nationals with a residence card are required by law to be enrolled in a health insurance programme.^4^ The free-access system allows patients to visit a hospital directly without referral from a family physician.^3^ Although the Japanese healthcare system has achieved better healthcare outcomes, such as long life expectancy and low infant mortality, it is facing two major challenges: financial sustainability of the system and a rapidly ageing population.^3^ Therefore, the MHLW has emphasised strengthening primary care.

A report on board certification by the MHLW included the competencies of family physicians such as comprehensiveness, continuity of care, and care for people with multimorbidities.^2^ However, this and other reports by the MHLW often do not cite peer-reviewed articles, but rather present arguments from the bureaucracy or an ‘expert’ panel. Also, a review about the Japanese healthcare system by the Organisation for Economic Co-operation and Development (OECD) emphasised a need for strengthening primary care.^5^ It indicates that primary care in Japan has been delivered by ‘semi-generalists/semi-specialists’, which are ‘physicians who leave hospital practice after an unspecified amount of time to set up as generalists (with no compulsory further training) in the community.’^5^ The OECD review insists on fostering primary care specialists to provide comprehensive and longitudinal care. To achieve this, the review recommended support for the creation of academic departments of family medicine, development of postgraduate training, introduction of rostering patients, capitation, and nurse-led primary care.^5^ Nevertheless, the review did not demonstrate any tangible target values such as minimum necessary budgets for fostering family physicians to support its recommendations.

The JMA, one of the biggest stakeholders in Japanese health care, had been cautious about the introduction of family medicine as a specialty, because they have considered that existing doctors in the JMA (semi-generalists/semi-specialists) can offer sufficient primary care without official training in family medicine.^6^ The JMA has also been apprehensive about the creation of a family medicine specialty since they believe it will lead to the introduction of capitation, which they assume will obstruct professional autonomy.^6^ Notably, the JMA has been gradually changing their cautious attitude; for example, the JMA has recently suggested a collaboration with certified family physicians in the future.^7^ However, as with the arguments from MHLW, these remarks by the JMA are not rigorously based on research evidence but their 'expert' opinions.

Another pertinent issue regarding the introduction of family medicine is the problem that few young doctors become family physicians in Japan.^8^ One reason is that family medicine is a new discipline in Japan and unfamiliar to medical students, patients, and specialist doctors. Thus, medical students usually do not have enough knowledge about family medicine when choosing their careers. Also, a poorly organised board certification system may contribute to the small numbers of applicants. The Japanese Medical Specialty Board was recently established to achieve high standards for physician specialty certifications with formal clinical training accredited by an independent third party.^4^ Many societies of specialists felt that their authoritative power was deprived by the Board.^4^ The Board changed its direction and guidelines in response to this criticism, and during this process, the application system and programme requirement changed repeatedly. Several websites reported that this reduction in Board governance may have negatively affected the number of applicants to family medicine.^9^ Moreover, other specialties may campaign against family medicine in order to attract young doctors to their own disciplines.^9^ Although these factors could be associated with young doctors’ decision making, the actual reasons of for why few doctors apply to become family physicians have not been adequately explored. Previous research from 2014 described several factors, such as lack of social recognition for family medicine, that may have a negative impact on a physician’s career choice.^10^ Further research is needed to explore these reasons.

As discussed, there is a lack of research evidence about the need for and roles of family medicine in Japan. As the current output of clinical research in primary care from Japan is very limited^11^ development of research capacity is a top priority and identifying high priority research topics is critical to achieve this. Relevant topics could be improving care transitions between primary and secondary care,^12^ identifying an ideal proportion of the healthcare budget to foster family physicians, exploring young physicians’ and specialists’ perspectives on family medicine, or discussing the role of family physicians in the Japanese healthcare setting. In other Asian countries such as China, medical universities have established family medicine departments and the government has offered financial support to build research capacity.^13^ Collaboration between the government and universities has been promoted in Malaysia.^13^ Such activities would be useful for Japanese family medicine.
